# Autologous, Non-Invasively Available Mesenchymal Stem Cells from the Outer Root Sheath of Hair Follicle Are Obtainable by Migration from Plucked Hair Follicles and Expandable in Scalable Amounts

**DOI:** 10.3390/cells9092069

**Published:** 2020-09-10

**Authors:** Hanluo Li, Federica Francesca Masieri, Marie Schneider, Tina Kottek, Sebastian Hahnel, Kensuke Yamauchi, Danilo Obradović, Jong-Keun Seon, Sook Jung Yun, Rubén A. Ferrer, Sandra Franz, Jan-Christoph Simon, Bernd Lethaus, Vuk Savković

**Affiliations:** 1 Department of Cranial Maxillofacial Plastic Surgery, University Clinic Leipzig, 04103 Leipzig, Germany; hanluo.li@medizin.uni-leipzig.de (H.L.); Tina.Kottek@medizin.uni-leipzig.de (T.K.); Bernd.Lethaus@medizin.uni-leipzig.de (B.L.); 2 School of (EAST) Engineering, Arts, Science & Technology, University of Suffolk, Ipswich, Suffolk IP41QJ, UK; F.Masieri@uos.ac.uk; 3 Clinic for Hematology, Cell Therapy and Hemostaseology, University Hospital Leipzig, 04103 Leipzig, Germany; Marie.Schneider@medizin.uni-leipzig.de; 4 Polyclinic for Dental Prosthetics and Material Sciences, University Hospital Leipzig, 04103 Leipzig, Germany; Sebastian.Hahnel@medizin.uni-leipzig.de; 5 Kensuke Yamauchi, Department of Oral & Maxillofacial Surgery, Tohoku University, Sendai 980-8577, Japan; yamaken29@gmail.com; 6 Leipzig Heart Center, 04289 Leipzig, Germany; daniloobradovic2@gmail.com; 7 Chonnam National University Hwasun Hospital, Hwasun-gun 58128, Korea; seonbell@chonnam.ac.kr (J.-K.S.); sjyun@chonnam.ac.kr (S.J.Y.); 8 Clinic for Dermatology, Venereology and Allergology, University Hospital Leipzig, 04103 Leipzig, Germany; RubenA.Ferrer@medizin.uni-leipzig.de (R.A.F.); sandra.franz@medizin.uni-leipzig.de (S.F.); Jan-Christoph.Simon@medizin.uni-leipzig.de (J.-C.S.)

**Keywords:** mesenchymal stem cells, hair follicle, regenerative medicine, non-invasive, cell therapy, adipose tissue, differentiation

## Abstract

Background: Regenerative therapies based on autologous mesenchymal stem cells (MSC) as well as stem cells in general are still facing an unmet need for non-invasive sampling, availability, and scalability. The only known adult source of autologous MSCs permanently available with no pain, discomfort, or infection risk is the outer root sheath of the hair follicle (ORS). Methods: This study presents a non-invasively-based method for isolating and expanding MSCs from the ORS (MSCORS) by means of cell migration and expansion in air–liquid culture. Results: The method yielded 5 million cells of pure MSCORS cultured in 35 days, thereby superseding prior art methods of culturing MSCs from hair follicles. MSCORS features corresponded to the International Society for Cell Therapy characterization panel for MSCs: adherence to plastic, proliferation, colony forming, expression of MSC-markers, and adipo-, osteo-, and chondro-differentiation capacity. Additionally, MSCORS displayed facilitated random-oriented migration and high proliferation, pronounced marker expression, extended endothelial and smooth muscle differentiation capacity, as well as a paracrine immunomodulatory effect on monocytes. MSCORS matched or even exceeded control adipose-derived MSCs in most of the assessed qualities. Conclusions: MSCORS qualify for a variety of autologous regenerative treatments of chronic disorders and prophylactic cryopreservation for purposes of acute treatments in personalized medicine.

## 1. Introduction

The bench-to-bedside concept in regenerative cell-based therapies has been gaining momentum. In this light, wellbeing of the patient is considered a matter of the final successful therapeutic output coupled with minimized disadvantages such as discomfort, trauma, risk of infection, sampling amount, and aberrant immune response as well as with maximized harvest and constant availability of autologous material, relieved of ethical challenges. Meeting these needs and incorporating them into regulatory requirements for cell therapies adds to their complexity [1,2,3].

Adult stem cells could present a solution to these demands, as prêt-à-porter and available from several human tissues. Nevertheless, they are by rule obtainable by invasive procedures from secluded niches, still limited in harvesting and with low scalability [1].

Adult Mesenchymal Stem Cells (MSC) are currently reemerging as a major type of stem cell for cell therapies due to their capacity for tissue replacement [4], trophic and immunomodulatory paracrine effects [5], also achievable by secretome [6]. Additionally, MSCs are immune-privileged, virtually non-tumorigenic, and void of ethical issues. Moreover, a relatively recent position statement by the International Society for Cell and Gene Therapy (ISCT) Mesenchymal Stromal Cell (ISCT MSC) committee has reiterated the criteria for defining MSCs [7] and pointed out that the ‘Mesenchymal Stem Cell’ nomenclature should be used in conjunction with a clear reference to the tissue source origin, together with providing a robust cell characterization, also in light of anticipated clinical applications [8].

Although MSCs are recognized as a somatic stem cell base for clinical applications [9], they are no exception to the aforementioned limitations. While they can be routinely sampled from human bone marrow [10], peripheral blood [11], adipose tissue [12,13], lung [14], wisdom teeth [15], deciduous teeth [16], synovial fluid [17], fallopian tubes [18] placenta [19], and umbilical cord blood [20], the only known source of non-invasively and stably available autologous MSCs, as well as stem cells in general, is the outer root sheath (ORS) of hair follicle [21].

The ORS is an envelope that surrounds the inner root sheath of cells and the hair shaft [22]. It contains a heterogeneous pool of cells, including stem cells characterized by one of the highest developmental potency in adults, neural crest-like stem cells (NCSC-like cells) [23,24,25], and Lgr6+ [26,27] and their downstream progeny—neural cell line, ectodermal and mesenchymal stem cells, skin stem cells, skin progenitors, and differentiated skin cells. The evolutionary role of this ‘stem cell zoo’, as it is sometimes called [28], is to regenerate the skin [27,29,30] and adjacent tissues [23,24,31]. This small, putative mini-organ, therefore, presents a life-long, non-invasively available autologous source of scalable stem cells for personalized regenerative therapies.

Several procedures for cultivation of MSCs from the ORS have been reported [32,33,34,35,36]. The majority of the prior art methods utilized dissected human scalp tissue to extract hair follicles, only available by highly invasive sampling. Three of those methods were based on an outgrowth of cells from non-invasively plucked hair follicles submerged in cell medium onto cell culture plastic polystyrene, with additional efforts to affix the follicles and allow migration onto the support. The cells cultured by these means were later characterized as MSCs and further aspects of their reprogramming, interface with biocompatible materials, and downstream applications involving growth factors were addressed [33,34,35]. All approaches have faced issues in reproducibility, scalability, and purity, at times with incomplete MSC marker phenotyping [33,34,35].

This study presents a reproducible method of culturing MSCs from the ORS (further referred to as MSCORS) in therapy-relevant numbers and in early passages. It further focuses on the full characterization, method advantages, the migratory capacity, and exquisite features of the non-invasively obtained MSCORS in comparison to moderate-invasively obtainable adipose-derived MSCs (ADMSC) [37]. Along with the possibilities of culturing and upscaling MSCORS, their cell motility, metabolic activity, and immunomodulatory properties are particularly interesting both from fundamental and applicative aspects of MSC biology. We aimed at looking into these features, beginning with mitochondrial activity, usually correlated to cell proliferation and migratory propensity, as a common landmark for cell responsiveness to stimulation towards paracrine-mediated anti-inflammatory effects, a basis for chemotaxis, and ultimately regenerative potential [38,39].

## 2. Materials and Methods

All the experiments were institutionally approved by the Ethical Committee of Medical Faculty, University of Leipzig (427/16-ek). Experimental procedures were performed according to the International Society for Stem Cell Research (ISSCR) guidelines. Human hair follicles were plucked from 5 healthy 20–48 years old donors (*n* = 5) in 2 hair-plucking sessions, yielding 60 anagen hairs per session. The samples of each donor were used to perform 3 independent biological experiments in 3 technical replicates. Adipose tissue was obtained from donors (*n* = 5) who underwent general trauma surgery at the Department of Dermatology, Venerology and Allergology at the Leipzig University Clinic. Written informed consent was obtained from all donors. For samples from each donor, the following experiments were replicated 3 times with 3 experimental repetitions.

### 2.1. Isolation and Culture of MSCORS

Human hair follicles were non-invasively epilated from the occipital region of donor’s scalp (*n* = 5, 2 sampling sessions). Sixty hairs were plucked per sampling, and hairs in the anagen phase were selected upon the presence of ORS. The hair follicles were immersed in the washing medium (Table S1) for 2 h at room temperature. Hair shafts were shortened to 2–5 mm length and a proximal part of the follicle was excised in order to eliminate the dermal carry-over. The shortened follicles were extensively rinsed 10 times in 10 mL washing medium for 5 min. Subsequently, the hair follicles were digested with 5 mg/mL collagenase X for 12 min, followed with FBS neutralization and brief rinsing. The hair follicles were placed onto a 0.4 μm-pore polystyrene mesh of a 6-well plate Transwell membrane insert (Corning Inc., New York, NY, USA), with the lower chamber filled with MSCORS Isolation Medium (Table S1), hence forming an air–liquid interface setup. Hair follicles were further incubated under hypoxic conditions (5% O_2_, 5% CO_2_) at 37 °C. After 7 days, the cells started to migrate from the hair follicle ORS into the Transwell membrane and formed a monolayer within 14 to 24 days. At 90% confluence, the upper chamber was filled with MSCORS Isolation Medium and further incubated for 48 h under hypoxic conditions.

To harvest the ORS cells, the cells were harvested from the Transwell membrane using 0.04%/0.03% Trypsin/EDTA. Trypsin was applied for 5 min 2–3 times until full detachment. After FBS neutralization, cell suspension from each 6-well insert was centrifuged, resuspended, pressed through a 70 μm cell strainer, and subcultured onto 2 wells of the 6-well plate. Ultimately, the cells from all wells of a particular donor were pooled.

After 24 h attachment, the non-adherent cells were withdrawn by PBS rinsing and the adherent cells were further cultivated for the next 5 days in the expansion medium. Subsequently, the cells were subcultured in a T75 flask with the MSCORS expansion medium (Table S1), reseeded at 10,000 cells per cm^2^, and labeled as passage 1 (P1) cells. Cells were further subcultured at 90% confluence in the passage ratio of 1:2 or 1:3.

### 2.2. Isolation and Culture of Adipose Derived MSCs (ADMSCs)

The adipose tissue of 5 donors (*n* = 5) was rinsed in PBS containing penicillin/streptomycin, sliced into 2 × 2 mm pieces, followed by digestion using 2 mg/mL collagenase X in MSCORS washing medium at 37 °C for 4 h with intermittent shaking. After FBS neutralization, digested adipose tissue was intensively vortexed and centrifuged for 10 min at 600 g at 20 °C. The cell-containing pellet was washed twice in PBS and pressed through a 100 μm nylon cell strainer to obtain a single-cell suspension. All cells were seeded into 75 cm^2^ flasks in the expansion medium (Table S1).

### 2.3. Determination of Cell Count and Cell Mitochondrial Activity

MSCORS and ADMSC (*n* = 5) were seeded with the same density of 1.2 × 10^4^ cells/cm^2^ in P0, and subcultured to the next passage in the ratio of 1:2 upon reaching 90% confluence. The cell count and mitochondrial activity were assessed in each passage.

Mitochondrial dehydrogenase activity was assessed using the chromogenic WST-1 cell proliferation assay (Roche Ltd., Basel, Switzerland). Briefly, 1 × 10^4^ cells/well were seeded in a flat-bottom 96-well plate and left to attach for 12 h in hypoxic conditions at 37 °C. WST-1 reagent was added into the medium at a ratio 1:10 and incubated for 60 min at 37 °C. The absorbance was measured at 450 nm, against a reference wavelength of 620 nm with a Synergy H1 spectrophotometry plate reader (BioTek Inc., Winooski, VT, USA). 

### 2.4. Determination of Cell Movement: Live Cell Imaging Time Lapse

Six-thousands cells of MSCORS and ADMSC (*n* = 5) were seeded on 4-well chamber slides (ibidi GmbH, Planegg, Germany), incubated in hypoxic conditions at 37 °C for 24 h. The attached cells were photo documented in a Keyence BZ-9000 Live Cell Imaging System (Keyence GmbH, Neu-Isenburg, DE, USA) at 10-min interval over a period of 24 h. In total, 145 images were imported as temporal stacks to the ImageJ version 1.53a software (https://imagej.nih.gov/ij/) and analyzed with the ImageJ Chemotaxis/Migration tool (https://ibidi.com/img/cms/products/software/chemotaxis_tool). To quantify cell movement, manual tracking of 15 single cells per stack, 3 stacks per donor, was performed over time by determining their position in each frame of the image stack. The tracking files (tab-delimited text) were imported into the Chemotaxis and Migration tool for further analysis of the accumulated and Euclidean distances, velocity, and directionality.

### 2.5. Determination of Gene Expression: Quantitative Real-Time Polymerase Chain Reaction (qRT-PCR)

Total RNA was isolated from the cells at passages P2–P5 using Qiazol Lysis Reagent (Qiagen, Hilden, Germany) and RNeasy Plus Universal Kit (Qiagen, Hilden, Germany) (*n* = 5). Using the QuantiTect Reverse Transcription Kit (Qiagen, Hilden, Germany), 1 μg of total RNA was reverse transcribed into cDNA. Target gene expression was assessed in triplicate via qRT-PCR using the QuantiFast SYBR^®^ Green PCR Kit (Qiagen, Hilden, Germany). Primers were designed using Primer3 web version 4.1.0 (60 °C annealing temperature) and manufactured by Invitrogen. The primer sequences are specified in Table S2.

A total of 5–50 ng cDNA was used for each 20 μL reaction. Thermal cycling was carried out at 95 °C for 60 s, followed by 40 cycles of 95 °C for 10 s, and 60 °C for 30 s. The expression level of analyzed genes was normalized to the mean expression of hypoxanthine-guanine phosphoribosyltransferase (HPRT) and calculated by a comparative 2^−ΔΔCt^ method of relative quantification using 7500 Software v2.3 (Qiagen, Hilden, Germany). 

### 2.6. Determination of Cell Surface Marker: Fluorescence-Activated Cell Sorting (FACS)

FACS analysis was performed at passages P2–P5 in triplicate using the BD Stemflow™ hMSC Analysis Kit (BD Biosciences, San Jose, CA, USA) (*n* = 5). MSCORS and ADMSC were trypsinized, rinsed with FACS buffer (0.5% BSA and 0.1% NaN3 in DPBS), and incubated for 40 min at 4 °C with fluorescently conjugated antibodies CD44 (Clone G44-26; BD Biosciences, San Jose, CA, USA), CD73 (Clone AD2; BD Biosciences, San Jose, CA, USA), CD90 (Clone 5E10; BD Biosciences, San Jose, CA, USA), CD105 (Clone 266; BD Biosciences, San Jose, CA, USA), and an MSC negative marker comprised of CD19 (Clone HIB19; BD Biosciences, San Jose, CA, USA), CD34 (Clone 581; BD Biosciences, San Jose, CA, USA), CD45 (Clone HI30; BD Biosciences, San Jose, CA, USA), HLA-DR (Clone G46-6; BD Biosciences, San Jose, CA, USA), and 11B (Clone CRF44; BD Biosciences, San Jose, CA, USA). Labeled cells were washed, resuspended in FASC buffer, and analyzed using the BD FACS Canto II device (BD Biosciences, San Jose, CA, USA), BD FACSDiva™ and FlowJo 7.6 software. Gating was determined against antibody fluorochrome isotype controls.

### 2.7. Determination of Cell Surface Marker and Differentiation Markers: Immunofluorescence Staining

MSCORS and ADMSC (*n* = 5) in passages 2–5 were trypsinized, seeded onto 8-well chambered slides (ThermoFisher Inc., Waltham, MA, USA), and left to adhere for 24 h. Sequentially, cells were fixed with 4% paraformaldehyde (PFA) and blocked with 10% normal goat serum (ThermoFisher Inc., Waltham, MA, USA). For ORS cell monolayer immunostainings, a hair follicle with an ORS cell layer was both cultivated and fixed on the Transwell membrane (Corning Inc., New York, NY, USA). For chondrogenic pellet immunostainings, the pellet was fixed, embedded in paraffin, and sectioned in 5 μm thickness, deparaffinized, rehydrated, and blocked with the 10% normal goat serum.

Targeted primary antibodies, Nestin (clone 10C2; ThermoFisher Inc., Waltham, MA, USA), CD44 (Clone G44-26; BD Biosciences, San Jose, CA, USA), CD90 (Clone 5E10; Sigma-Aldrich GmbH, Schnelldorf, Germany), STRO-1 (Clone STRO-1; Sigma-Aldrich GmbH, Schnelldorf, Germany), Nestin (Clone 10C2; ThermoFisher Inc., Waltham, MA, USA), Collagen II (Clone COL-II; Abcam Plc, Cambridge, UK), CD31 (Clone P2B1 Abcam Plc, Cambridge, MA, USA), and alpha smooth muscle actin (αSMA, Abcam Plc, Cambridge, UK) diluted 1:200 in blocking buffer were incubated with cells overnight at 4 °C, washed, incubated with secondary antibody AlexaFluor^®^ 594-conjugated goat anti-mouse IgG (1.0 mg/mL) (Life Technologies, ThermoFisher Inc., Waltham, MA, USA), and counterstained with 4′, 6-diamidino-2-phenylindole (DAPI).

### 2.8. Osteogenic Differentiation

MSCORS and ADMSC (*n* = 5) were seeded at 2 × 10^4^ cells/cm^2^ density and incubated in osteogenic medium (Table S1) for 4 weeks in hypoxic conditions at 37 °C. Extracellular calcium deposits were stained by Alizarin Red Staining using 2% Alizarin Red Solution. The activity of intracellular alkaline phosphatase (ALP) was detected using Sigma FAST^TM^BCIP/NBT substrate (Sigma-Aldrich GmbH, Schnelldorf, Germany).

### 2.9. Chondrogenic Differentiation

MSCORS and ADMSC (*n* = 5), resuspended in the MSCORS chondrogenic medium at density 2.5 × 10^5^ cells in 1ml, were (Table S1) centrifuged at 800 g for 10 min in a 15 mL conical tube and incubated with a loosened lid in hypoxic conditions at 37 °C for 4 weeks. The cartilaginous pellet was fixed in 4% PFA overnight, embedded in the paraffin, and sectioned in 2 μm thickness for histological staining. Hematoxylin and Eosin (H&E) staining was carried out using Mayer’s Hematoxylin and Eosin (Carl Roth GmbH, Karlsruhe, Germany). Proteoglycans were stained with 1% Alcian Blue Solution (Sigma-Aldrich GmbH, Schnelldorf, Germany) for 30 min and counterstained using Nuclear Fast Red solution (Sigma-Aldrich GmbH, Schnelldorf, Germany).

### 2.10. Adipogenic Differentiation

MSCORS and ADMSC (*n* = 5) were seeded into 24 well plates at a seeding density of 0.67 × 10^4^ cells/cm^2^ and incubated in StemPro™ Adipogenesis Differentiation Medium (Gibco, ThermoFisher Inc, Waltham, MA, USA) for 3 weeks at 5% CO_2_ at 37 °C. Intracellular lipid vesicles were stained using Oil Red O (Sigma-Aldrich GmbH, Schnelldorf, Germany). 

### 2.11. Endothelial Differentiation

MSCORS and ADMSC (*n* = 5) before P5 were seeded into a 6-well plate at a density of 2.5 × 10^4^ cells per cm^2^ endothelial medium (Table 1) and differentiated for 4 weeks in endothelial medium (Table S1) at 5% O_2_, 5% CO_2_, 37 °C. The differentiated cells were fixed in 4% PFA and stained with primary antibody CD31. To assess endothelial differentiation, the tube forming assay was performed using Corning^®^ Matrigel^®^ Matrix (Corning Inc., New York, NY, USA). Briefly, 100 μL of Matrigel was thawed in 4 °C and coated onto a 48-well plate. The differentiated endothelial cells were dissociated and seeded onto the Matrigel membrane at a density of 6 × 10^4^ cells per well and were incubated for 8 h in hypoxic conditions at 37 °C. The proangiogenic tubes formed by endothelial differentiated cells were stained using Live/Dead Assay (ThermoFisher Inc., Waltham, MA, USA) containing Calcein AM and Propidium Iodide (PI). Angiogenesis with live/dead fluorescence were imaged by a Keyence BZ-9000 Fluorescence Microscope.

### 2.12. Smooth Muscle Differentiation

MSCORS and ADMSC (*n* = 5) were seeded into a 6-well plate at a density of 2.5 × 10^4^ cells/cm^2^ and incubated in smooth muscle medium (Table S1) for 21 days at 5% O_2_, 5% CO_2_, 37 °C. The presence of alpha smooth muscle actin (αSMA) was determined by immunostaining.

### 2.13. Determination of Cell Immunomodulatory Effects

Human CD14+ PBMNC were freshly isolated from the whole blood of healthy individuals (*n* = 3) using the CD14+ MicroBeads Isolation Kit (Miltenyi Biotec Inc., Bergisch Gladbach, Germany). CD14+ PBMNC were differentiated in the presence of 50ng/mL granulocyte macrophage–colony stimulating factor (GM-CSF) for 6 days towards M1-like macrophages followed by another 24 h stimulation of 100 ng/mL lipopolysaccharide (LPS) to provoke their inflammatory activation. Throughout differentiation and activation, 2 × 10^5^ CD14+ PBMNC were co-cultivated with 2 × 10^4^ MSCORS (*n* = 3) and ADMSC (*n* = 3) at 37 °C. After differentiation, activated macrophages from CD14 + PBMNC were characterized by flow cytometry using CD163 antibody for the alternatively activated M2 phenotype. The levels of released cytokines in cell-free supernatants were quantified using the ELISA kit with antibodies against IL-12p40 (BD Biosciences, San Jose, CA, USA), tumor necrosis factor alpha (TNF-α), and IL-10 (eBioscience Inc., San Diego, CA, USA), according to the manufacturer’s instructions.

### 2.14. Statistical Analysis

Statistical evaluation of the quantitative results was done by an unpaired *t*-test or nonparametric Mann–Whitney test. Normal distribution and the homogeneity of variance of datasets were checked by a Shapiro–Wilk normality test and F-Test. *p* values ≤ 0.05 were regarded as statistically significant.

## 3. Results

### 3.1. MSCORS Isolation by Migration and Adherent Culture

**Figure 1:**
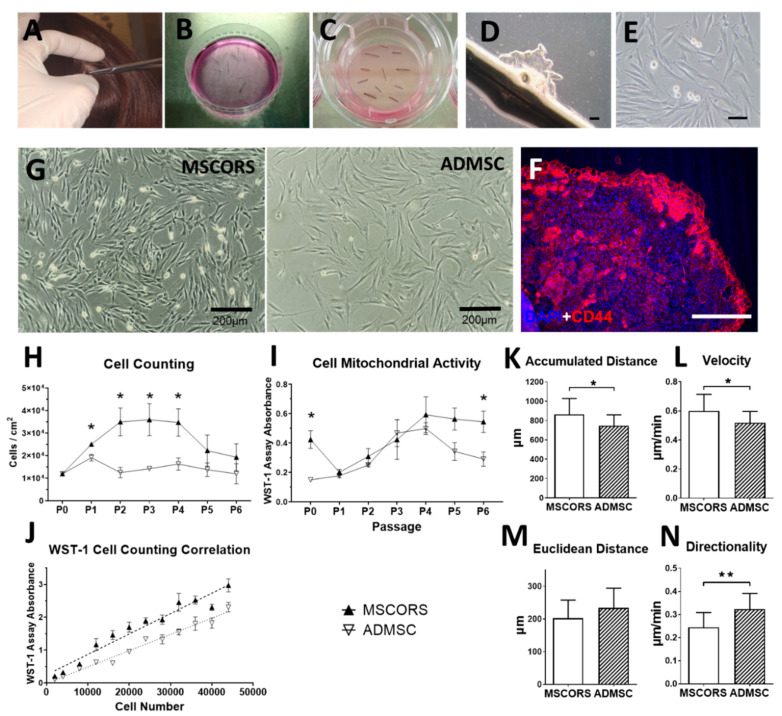
Isolation and cultivation of MSCORS compared to ADMSC. Hair follicles were plucked from the human scalp of five healthy donors (*n* = 5) in double harvesting sessions of 60 hairs per session (**A**), shortened distally and proximally, washed (**B**), and explanted on a Transwell membrane (**C**). From day 3 to day 24, cells migrated out from the ORS and formed a cell monolayer with a colony-like structure (**D**). Upon reaching confluence, the ORS cell monolayer was subcultured in flasks (**E**). (**F**): The ORS cell monolayer was positive for CD44 (red) with intensive expression at the outer ORS edge. (**G**): After subcultured in a flask, MSCORS displayed an MSC-like morphology similar to ADMSC. Serial cell subcultures of MSCORS and ADMSC over 1–6 passages, with cell counting (**H**) and projected cell yield (**I**) in each passage, correlated with cell mitochondrial activity (**J**). Cell migration of MSCORS and ADMSC, including accumulated distance (**K**), velocity (**L**), Euclidean distance (**M**), and directionality (**N**). Data are shown as mean ± SD (* *p* < 0.05, ** *p* < 0.01) (*n* = 5), with unpaired *t*-test or nonparametric Mann–Whitney test. Scale bar corresponds to 200 μm; magnification (**A**) 2×, (**E**,**F**) 10×, and (**G**) 4×.

Typically, 60 hair follicles in the anagen phase were plucked per isolation, summing up to 21.45 ± 12.16 mg tissue weight due to individual differences. The percentage of hair follicles of the five donors in three biological experiments that yielded migrating cells was 70.21 ± 16.64%. Within 25 days of air–liquid culture, cells migrated from the ORS and proliferated on Transwell polystyrene mesh, forming a nearly confluent cell monolayer. The migrating monolayer was visible by microscope and evident by immunostaining of CD44, Nestin, and Stro-1, with a clearly discernible CD44 fluorescence signal at the leading edge and along the migration routes of the MSCORS (Figure 1F, Figure S1B–D, respectively).

After 19 ± 6 days of air-to-liquid interface culture, MSCORS were detached from the polystyrene membrane and transferred to the routine cell culture on adherent plastic. The adherent culture on plastic culture yielded dendritic, elongated, spindle-shaped adherent cells, as shown in Figure 1G. The cells were successfully maintained in culture over six passages without displaying typical visible morphological signs of cell senescence such as hypertrophic size, morphological changes, enlarged cytoplasm, or abnormal nuclear shape [40]. The overall diameter in cell suspension was 20.52 ± 0.59 μm in MSCORS, 20.95 ± 1.37 μm in ADMSC, and it remained comparable in all passages.

Hereby, in total, 50 million cells in P2 were obtained in 54 days from follicle extraction using the 60-follicle setup. The cell numbers per passage are shown in Table 1.

Cell attachment and expansion of the initial monolayer, referred to as passage 0 (P0), required 14 ± 5 days of culture, yielding averagely 83,408 ± 44,650 proliferating cells from one hair follicle in P0, regardless of individual differences. Passage 1 (P1) required 10 ± 6 days and produced 365,041 ± 133,188 cells per hair follicle. Passage 2 (P2) required 11 ± 5 days and yielded 838,576 ± 463,160 cells per hair follicle.

### 3.2. Cell Proliferation and Mitochondrial Activity

To determine cell proliferation and viability, the cultivation of MSCORS was compared with ADMSC from P0 to P5. MSCORS and ADMSCs were cultivated from P0 with the same cell seeding density 1.2 × 10^4^ cells/cm^2^ and subcultured in ratio of 1:2 upon reaching 90% confluence. As shown in Figure 1H, with the same cell seeding density in P0, MSCORS yielded a noticeably higher cell count than ADMSC in P1 to P4 (*p* < 0.05), by 30.96% in P1, 179.33% in P2, 152.81% in P3, and 112.65% in P4. The cell count of MSCORS elevated from P1, peaked in P3, and declined thereafter, whereas ADMSC yielded similar cell numbers in each passage.

Mitochondrial activity was quantified using the WST-1 assay, and it showed close correlation to MSCORS and ADMSC cell count in Figure 1J. The mitochondrial activity increased from P1 onward, peaked at P4, and declined thereafter (Figure 1I), which followed the cell count trend with a one-passage shift. Mitochondrial activity was measured using the same number of seeded cells. In P0 and P5, the mitochondrial activity of MSCORS was significantly higher than that of ADMSC (183.20%, *p* < 0.05 and 64.37% higher, *p* < 0.05, respectively) and at P6 (87.34% higher, *p* < 0.05), whereas it was comparable to the ADMSC in P1, P2, P3, and P4.

### 3.3. Cell Motility and Migration Parameters

Accumulated distance and the overall migration velocity charted by MSCORS were significantly higher than that of the ADMSCs by 8.32% (Figure 1K, *p* = 0.0381) and 10.48% (Figure 1L, *p* = 0. 0416), respectively. MSCORS displayed non-significantly lower Euclidian distance by 8.73% and significantly lower directionality by 17.88% (Mann–Whitney test, *p* = 0.0039) than ADMSCs, respectively (Figure 1M,N).

### 3.4. Gene Expression

**Figure 2:**
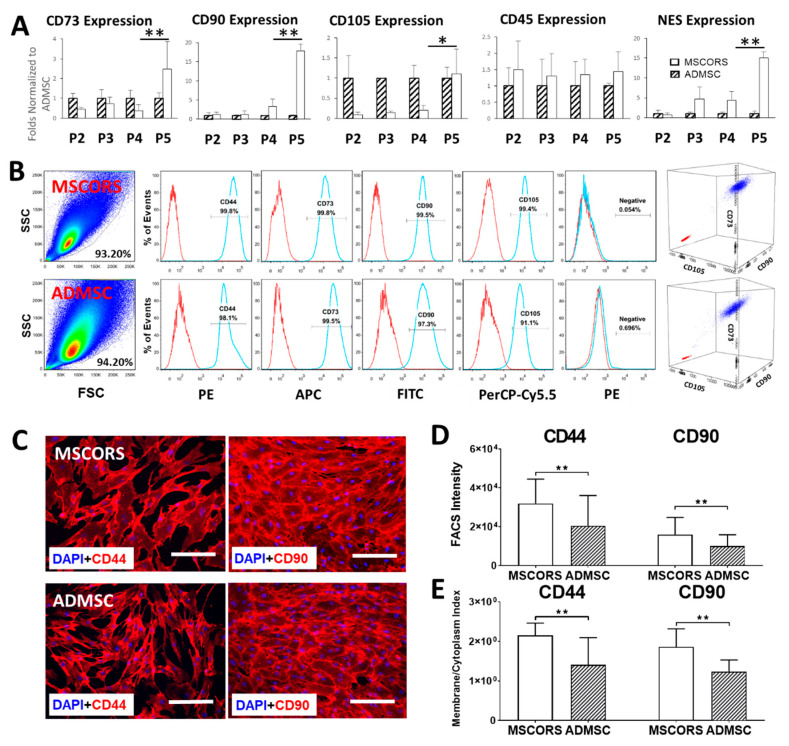
Phenotype of MSCORS compared with ADMSC. (**A**): qRT-PCR showing the relative gene expression of *CD73*, *CD90*, *CD105*, *CD45*, and *NES* of MSCORS against ADMSC from passage 2 to passage 5 (*n* = 5). *HTRP-1* was used as the internal reference. (**B**): Flow cytometer analysis of surface marker expression on MSCORS and ADMSC. Cells from passage 3 to 5 were labeled with antibodies against human antigens CD44, CD73, CD90, CD105, and MSC-negative marker combination (CD45/CD34/CD11b/CD19/HLA-DR). Representative plot graphs and histograms are shown with antibodies (blue) and isotype control (red). (**C**): Expression of CD44 and CD90 in MSCORS and ADMSC (*n* = 5). CD44 and CD90 display varied overall intensity and different subcellular distribution. (**D**) Expression of CD44 and CD90 analyzed from FACS signal intensity. (**E**) Membrane/cytoplasm index of CD44 and CD90 expression intensity in MSCORS and ADMSC. Data are shown as mean ± SD (* *p* < 0.05, ** *p* < 0.01) (*n* = 5). Scale bar corresponds to 200 μm; magnification (**C**) 10×.

MSCORS expressed typical MSC marker genes *CD73*, *CD90*, *CD105*, *CD45*, and *NES* along four analyzed passages until P5 (Figure 2A). The relative marker gene expression of MSCORS and comparative gene expression normalized to ADMSC was increasingly elevated throughout the cultivation period, except for *CD45*. In particular, MSC-positive markers *NT5E* (*CD73*), *THY1* (*CD90*), *ENG* (*CD105*), and additionally, *NES* (*Nestin*) were increased in higher passages with expression peaks of the *CD73*, *CD90*, *CD105*, and *NES* at P5 in MSCORS, reaching significantly higher values in comparison to P4 (*p* = 0.0078, *p* = 0.00368, *p* = 0.02753, *p* = 0.0095, respectively), exceeding those of the ADMSCs (*CD73* by 115.86%, *p*= 0.18; *CD90* by 16.91 folds, *p* = 0.0057; *CD105* by 10.99%, *p* = 0.44; *NES* by 20.52 folds, *p* = 0.0072). *CD45* expression in both MSCORS and ADMSCs maintained steady low levels.

### 3.5. MSC Surface Marker Expression

FACS analysis (Figure 2B) revealed an aggregated, homogenous, morphologically compact population of MSCORS compared to a more diffuse population of ADMSCs in the forward scatter vs. side scatter plot. The analysis of CD44, CD73, CD90, and CD105 expression revealed a correct expression pattern in both single and triple staining for MSCORS and ADMSCs, with over 99% positive cells for all markers in MSCORS versus at least 91% positive cells in ADMSCs. Quantitation of fluorescent signals in FACS (Figure 2D) revealed significantly higher expression of CD44 and CD90 in MSCORS than in ADMSC (by 55.55% in CD44, *p* < 0.01; by 57.51% in CD90, *p* < 0.01). Both MSCORS and ADMSCs displayed undetectable levels of negative markers CD45, CD34, CD11b, CD19, and HLA-DR. 

### 3.6. Protein Expression and Subcellular Distribution

MSCORS expressed CD44, CD73, CD90, CD105, and Nestin, exhibiting a clear subcellular distribution pattern of the markers. Compared to the distribution pattern of ADMSCs, the subcellular distribution of CD44 and CD90 was more distinct in MSCORS; MSCORS showed signals of CD44 and CD90 expression along the membrane edge of the cell more intense when compared to that of the intracellular cytoplasm (Figure 2C), whereas in ADMSC, the fluorescent signals in the membrane and cytoplasm were comparable in intensity. Quantification of the subcellular distribution performed for CD44 and CD90 revealed a statistically significant higher membrane/cytoplasm index in MSCORS than in ADMSCs by 55.16% in CD44 and by 50.19% in CD90 (Figure 2E).

### 3.7. Differentiation Potentials

As an essential part of the ISCT characterization panel for MSC classification, capacity for adipo-differentiation, chondro-differentiation, and osteo-differentiation was validated in MSCORS with ADMSCs as a control. Additionally, capacity to differentiate into smooth muscle and endothelial cells has also been demonstrated.

**Figure 3:**
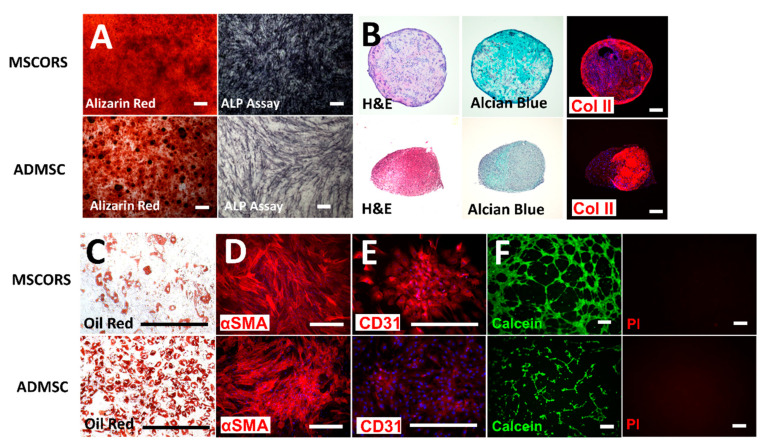
Differentiation competences of MSCORS and ADMSC. (**A**): Osteogenic differentiation for 28 days. The extracellular CaP deposition in MSCORS and ADMSC (*n* = 5) was displayed by Alizarin Red staining, and intracellular ALP activity was visualized by BCIP/NBT. (**B**): Chondrogenic differentiations of MSCORS and ADMSC for 28 days by pellet culture (*n* = 5). Chondrogenesis was assessed via H&E and Alcian Blue for proteoglycan content. Type II collagen was identified via immunostaining. (**C**): Adipogenic differentiation of MSCORS and ADMSC for 21 days (*n* = 5). The intracellular lipid vesicles were detected by Oil Red O staining. (**D**): Smooth muscle differentiation for 21 days was evaluated via αSMA immunostaining (*n* = 5). (**E**): Endothelial differentiation of MSCORS and ADMSC for 21 days detected via immunostaining for CD31 (*n* = 5). (**F**): The potential for vascular anastomosis was investigated using a tube forming assay and highlighted by Live/Dead staining of Calcein AM and PI, respectively, displayed in separate channels (*n* = 5). Immunostaining was counterstained with DAPI. Scale bar corresponds to 200 μm; magnification (**A**) 4×, (**B**) 4×, (**C**) 20×, and (**D**) 10×, 20×, and 4×.

After 28 days of osteogenic differentiation, MSCORS and ADMSCs produced clearly visible calcium phosphate (CaP) vesicles on top of the cell layers that were stained using Alizarin Red, as shown in Figure 3A. The presence of ALP was also determined by the ALP assay with the appearance of a dark blue coloration. These indicated a clear endpoint outcome of osteogenic differentiation in both MSCORS and ADMSC, resulting in a functional osteoblast and mineralized matrix with mineral deposits.

MSCORS and ADMSCs directed to chondro-differentiation for 21 days in pellet culture confirmed chondrogenesis (Figure 3B). Microscopy analysis revealed hypertrophic chondrocytes with better compact structure and larger size of chondrogenic pellet in MSCORS than in the ADMSC. The loosened structure observed in the ADMSC-generated chondrogenic pellet indicated arguably a lower level of deposited extracellular matrix. The microscopic analysis of the pellet revealed a large structure and an intense Alcian blue staining uptake, showing a remarkable ECM deposition and glycosaminoglycans accumulation in MSCORS-differentiated cells. The expression of type II collagen, a marker of articular hyaline cartilage, was identified using immunostaining in both MSCORS and ADMSC.

Adipogenic conditions lead the MSCORS and ADMSCs to the endpoint adipocyte, as determined by a strong bright red coloration by Oil Red staining of the perinuclear lipid vesicles in the functionally differentiated adipocytes, which resulted in intensive bright red coloration (Figure 3C).

Differentiation of MSCORS and ADMSC into smooth muscle cells was demonstrated by immunostaining of the α-smooth muscle actin (α-SMA, Figure 3D).

MSCORS and ADMSC were subjected to endothelial differentiation for 28 days in the presence of VEGF and BMP-4. Endothelial differentiation was confirmed by immunostaining of endothelial marker CD31. The angiogenic functionality of the endothelial cells differentiated from MSCORS and ADMSC was also identified using a Matrigel^TM^-based tube forming assay, which revealed an anastomosis network formed by the differentiated MSCORS and ADMSC (Figure 3F).

### 3.8. Immunomodulatory Effects of MSCORS vs. ADMSC

**Figure 4:**
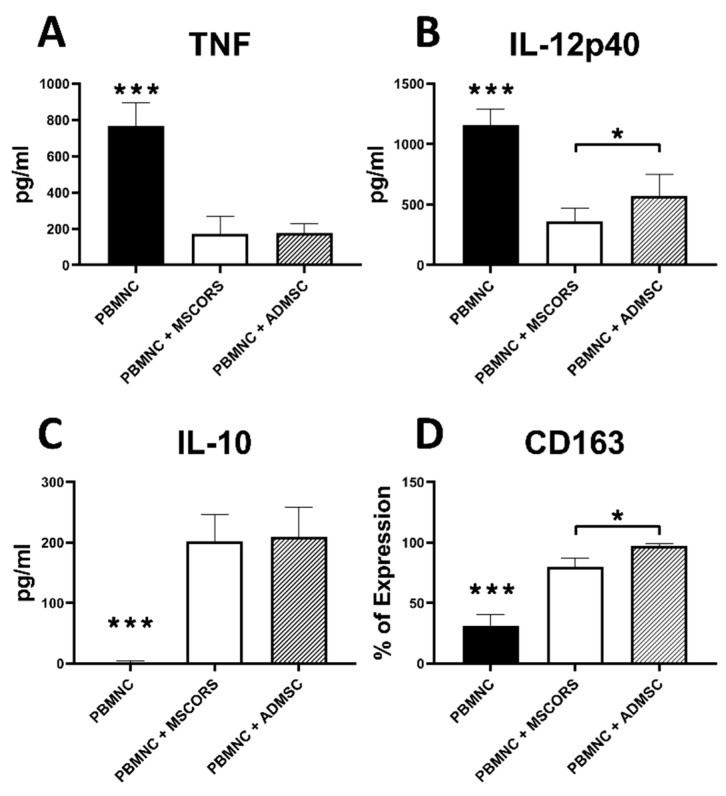
Immunomodulatory effects of MSCORS and ADMSC with stimulated PBMNC alone or in co-culture. MSCORS and ADMSC (*n* = 3) were pre-seeded and co-cultured with CD14 + PBMNC monocytes with 6 days GM-CSF stimulation and 1 day of treatment of LPS to obtain differentiated macrophages. CD14 + PBMNC-derived macrophages were counted by flow cytometry and levels of released cytokines including TNF-α, IL-12p40 and IL-10 were assessed by ELISA. (**A**) TNF-α, (**B**) IL-12p40, (**C**) IL-10, (**D**) CD163 expression. Data are depicted as mean ± SD (* *p* < 0.05, *** *p* < 0.001). *** asterisks above PBMNC signifies statistical difference versus all other groups.

Immunomodulation is a major function of MSCs, as recently postulated in the ISCT statement [8]. To investigate the immunomodulatory effect of MSCs, proinflammatory differentiation of monocytes using GM-CSF was employed in a co-culture with MSCs. Figure 4 shows that CD14 + PBMNC monocytes co-cultured with MSCORS or ADMSCs shifted the monocyte differentiation and polarization in proinflammatory settings towards macrophages with typical M2 functions. 

The level of soluble inflammatory mediators, TNF-α and IL-12p40, was significantly lowered. Co-culture with MSCORS decreased the level of TNF-α from 765.77 ± 106.23 to 173.29 ± 89.10 pg/mL, and of IL-12p40 from 1160.43 ± 104.16 to 358.85 ± 108.01 pg/mL compared with the non-cocultured monocytes, *p* < 0.001. The MSCORS effect on TNF-α was comparable to that of ADMSCs, whereas the release of IL-12p40 was significantly lower in MSCORS than the ADMSCs (reduction from 1160.43 ± 104.16pg/mL to 358.85 ± 108.01 pg/mL vs. 574.28 ± 167.12 pg/mL, respectively, *p* < 0.001) (Figure 4A,B).

Release of the anti-inflammatory cytokine IL-10 by monocytes was significantly increased in the co-culture with MSCORS and ADMSCs by 120-fold and 124-fold, respectively, compared with PBMNC monocytes without co-culture (*p* < 0.005) (Figure 4C). 

The expression of CD163, a M2 macrophage marker, was significantly increased after MSCs co-culture with monocyte-derived macrophages. Figure 4D displayed the expression of CD163 that was significantly upregulated 2.5-fold in co-culture with MSCORS and 3-fold in ADMSC compared with the differentiation without co-culture, respectively (*p* < 0.001). Here, ADMSCs exerted a significantly stronger effect on monocytes than MSCORS, increasing the percentage of CD163+ PBMNCs from 31.4 ± 7.32% to 97.18 ± 1.78% versus 79.88 ± 6.60%, respectively (*p* < 0.001).

## 4. Discussion

This study fulfills the yet unmet needs for non-invasively harvesting MSCs from the hair follicle ORS and for efficiently cultivating them into a pure culture of easily expanded stable cells. To date, this is the first method that enables culturing of MSCs from the hair follicle by means of cell migration, from the ORS onto the polystyrene mesh and their subsequent adherence, both well-known innate features of MSCs [41].

The combination of minimal sampling and a maximal output resulted in cell amounts scalable from 60 hair follicles to 5 million cells within 50 days of harvesting. To the best of our knowledge, the herein specified efficiency of the procedure upon the aforementioned minimal sampling has not been reached by other analogue methods to this day. Regarding their characteristics, MSCORS are not only equivalent to other MSCs according to the MSC characterization criteria of the International Society for Cell Therapy (ISCT) [7], but also, at least fully comparable to ADMSCs in terms of proliferation, metabolic activity, motility, expression profile, differentiating capacity, and immunomodulatory properties.

Culturing MSCs from hair follicles has been attempted previously and several prior art methods have been reported. Typically, the hair follicles in those works were isolated in an invasive fashion, either from operative remains of human scalp skin or from punch biopsies [42]. Microdissection of hair follicles from such skin rests has generally been the most commonly used method. This enabled preserving an intact bulge region of the follicle, which has greatly contributed to the accumulation of data relevant for ORS biology. Nevertheless, practical issues inherent to this design reduce its translational compatibility, not only the invasive approach but also a lengthy enzymatic digestion applied in order to loosen the tissue around the follicles prior to dissection, which may damage the cells in case of trypsin [43,44]. The prior art methods that used non-invasively plucked hair follicles leave room for optimization of efficiency, cell purity, and reproducibility [33,34,35]. They resulted in lower efficiency and cell yield compared with the MSCORS method in our reproduced experiments (Figure S2). 

Additionally, the characterization of the cells obtained by the comparable methods mentioned above characterized the MSC marker expression and differentiation profile only in part, leaving a possibility that the cells identified as hair follicle ORS-derived MSCs could in fact be residual dermal cells [33,34,35]. Similarly, a study and a patent application employed a microdissection of the dermal sheath [32] and withdrawal of the proximal hair follicle part in order to reduce the dermal carry over [42], followed by separation of the dermal sheet from the follicle by dissection. This procedure yielded a culture of non-bulbar dermal stem cells (NBDSC) that exhibited a partial overlap with an MSC expression profile.

The methodological steps used in this study circumvent the aforementioned challenges and improve the efficiency, yield, purity, and quality of the MSCs. These methodological improvements are discussed hereafter, along with the further advantages and translational potential of MSCORS. Sampling of hair follicles by the means of hair plucking is non-invasive and does not open way to infections. This further enables a clean-cut extraction of the follicle with minimal to no loss of the ORS tissue. The small amount of tissue that is being withdrawn from the skin can only be compared to that of inner tissue invasive biopsies such as retina or fallopian tube [18]. 

In case of dermal carry over, fibroblasts could quickly overgrow and suppress other less resilient cell types. By severing the proximal part of the follicle, a major source of dermal carry-over is removed [45]. Eliminating their carry-over proved to be a major step towards purity of the resulting culture [46]. Subsequent washing steps eliminate the crude impurities from the follicle surface, while the antibiotics and antimycotics effectively remove the resident bacteria and fungi [46]. 

Digesting the tissue in previously reported methods had two purposes: either to loosen the skin specimen and help in microdissecting the follicles from the skin or to completely digest the tissue and reach cell suspension. The collagenase X used in this study partially digests the majority of collagen types [47], loosening the extracellular matrix (ECM) and facilitating cell migration from the ORS onto the polystyrene mesh. Since collagenase is far less aggressive than trypsin, which is a non-specific protease used in other methods to digest the tissue, no cell damage occurs during the collagenase enzymatic digestion of the ECM [48]. After partially degrading the ECM, the cells could leave the ORS by their innate capability to migrate and populate the mesh of the suspended Transwell.

The air–liquid interface provided exposure to the hypoxic conditions favorable for expansion and plasticity as well as nutrition from the medium, simultaneously protecting the cells from desiccation [49]. The MSCORS were hereby provided space, nutrients, and humidity that favored their further migration and proliferation (Figure 1B–E). The cultivating conditions thereby helped the MSCORS move out of their native ORS niche and they conceivably were clearly able to very quickly adapt to the loss of the ORS niche and to the artificial in vitro conditions.

Here, bona fide MSCs have been obtained not only regarding their yield but their characteristics too. In this study, we have compared MSCORS to ADMSCs as the MSCs obtainable by liposuction, the least invasive harvesting method of stem cells known before the isolation of MSCORS from hair follicle ORS. MSCORS exhibited several features no less than comparable to those of ADMSCs in terms of isolation, expansion, and in vitro culture.

Firstly, it took 10mg of non-invasively sampled hair follicle tissue (obtained via plucking) to yield roughly 2.5 × 10^6^ MSCORS cells within 35 days of culture (Figure 1H,I, Table 1). Compared to the 25 g of medium-invasively sampled (liposuction) adipose tissue required to reach the same cell yield [21], it is clear that the method at hand provides a truly less invasive and extensive harvesting than the method of culturing ADMSCS or other known comparable methods.

We have shown that MSCORS are easier to isolate and cultivate than ADMSCs. Additionally, MSCORS exhibited more intensive proliferation (Figure 1H), comparable mitochondrial activity (Figure 1I,J), a moderately higher random cell motility (Figure 1K,L,N, more pronounced expression profiles of the MSC markers (Figure 2A,B), and a more demarcated subcellular distribution of strongly expressed CD44 and CD90 markers than ADMSCs (Figure 2B,C), reflected in their somatic/membrane index and relative fluorescence intensities in FACS plots (Figure 2D,E). Marker gene expression in MSCORS did generally intensify towards passage 5 (Figure 2A). All of the above makes MSCORS very versatile in terms of cultivation, providing maintained and even increased cell fitness over a high number of passages. This increases amplification and cryopreservation opportunities, without quality loss, making MSCORS at least comparable candidate for regenerative therapies when compared to ADMSCs, with a clear advantage of being harvested in an absolutely non-invasive way.

The proliferative propensity and prompt migration of the MSCORS may hint towards a hypothesis of a differentiation status other than that of the MSCs or even of transiently proliferating progenitors; however, we see this as unlikely in light of the shown MSC marker gene expression dynamics and absence of visible morphological senescence indicators (Figure 1E,G).

The migrating abilities of MSCORS fit well into the historically verified regenerative activity of the ORS stem cells or skin cells through migration towards the interfollicular injury sites [50] and they are concordant to the MSCORS cell motility in vitro demonstrated here (Figure 1K–N). The generally increased migrating activity of MSCORS enabled swift migration from the ORS to the PS mesh. We recognize this feature as the deciding factor for the efficiency of the method presented herein, by withdrawal of MSCORS from the ORS causing minimal-to-no stress and damage for the cells. The stringent, prevalently membrane-bound distribution of CD44 and CD90 in MSCORS, which are involved in cell–cell and cell–ECM interactions, cell adhesion, and migration [51,52], could be linked to their innate inclination towards migrating.

According to the minimal characterization requirements, MSCs should be capable of osteogenesis, chondrogenesis, and adipogenesis [7]. In this study, MSCORS not only exhibited triple mesodermal differentiation capacity but responded to additionally performed differentiations into endothelial cells and smooth muscle cells very efficiently. The link between high motility and extended differentiation capacity in MSCs has been previously reported [53] and the MSCORS high motility along with extended differentiation capacity are in accordance with it. This evident plasticity of MSCORS adds to their application potential for tissue replacement. 

MSCORS have exerted immunomodulatory paracrine effects in co-culture with monocytes, hereby showing a suppression of proinflammatory cytokines TNF-α and IL-12p40 and stimulating production of anti-inflammatory cytokines interleukine-10 (IL-10) and CD163 in M1 cells. The herein exhibited immunomodulation is also a known feature of MSCs [54] and we have therefore enriched the MSC characterization portfolio with that additional criterion. This display of immunomodulatory properties strengthened our hypothesis that the MSCORS can be reliably ranked among MSCs upon all known criteria and helped us identify an additional advantageous feature in terms of their translation potential.

The applicative potential of MSCORS is enhanced by their availability at almost any life phase, introducing a highly personalized angle through adjustments to the personal and professional lifestyle. Additionally, MSCORS fully retain their MSC phenotype, proliferative, and differentiation potential after cryopreservation (unpublished data). Cryopreserving MSCORS in therapy-relevant amounts as an off-the-shelf product would help overcome their major limitation—the long culturing time-frame—and enable a prompt reaction to acute injuries as well, analog to the general tendency of shortening the reaction time to treat acute degenerative disorders [55]. This particular option of shortening the bench-to-bedside interval builds a putative portfolio for MSCORS-based products in cell banking.

## 5. Conclusions

MSCORS are easily and reliably cultured based on their migratory capability and high proliferation capacity. Their high differentiation potential and stability in the in vitro primary culture for longer periods, yielding high numbers of cells, along with immunomodulatory effects and their plasticity, makes them optimal candidates for autologous regenerative therapies, be it tissue replacement, paracrine effects, as cells, or cell secretome applicable in chronic and potentially acute trauma and degenerative disorders.

## 6. Patents

The method of isolating MSCORS has been registered as an Invention Application with University of Leipzig (Erfindungsmeldung LP2) and as a patent application by EU Patent Office (PCT/EP2020/070027).

## Figures and Tables

**Table 1:** Average MSCORS yield before P2.

Number of Hair Follicles	Hair Weight (mg)	Cell Yield in P0 (Million Cells)	Cell Yield in P1 (Million Cells)	Cell Yield in P2 (Million Cells)
60	21.45 ± 12.17	5.00 ± 2.68	21.90 ± 9.46	50.31 ± 27.79

## Supplementary Materials

The following are available online at https://www.mdpi.com/2073-4409/9/9/2069/s1, Figure S1: Outgrowth process of MSCORS from the ORS and characterization of ORS cell monolayer, Figure S2: Comparative experiment of MSCORS isolation method vs. outgrowth methods in prior art literature, Table S1: Medium compositions for cell culture, Table S2: Primer sequences.
