# Supplementary material for: Autologous, Non-Invasively Available Mesenchymal Stem Cells from the Outer Root Sheath of Hair Follicle Are Obtainable by Migration from Plucked Hair Follicles and Expandable in Scalable Amounts
Source: Cells. 2020 Sep 10;9(9):2069. doi: 10.3390/cells9092069 (PMC7564264; doi:10.3390/cells9092069)
Supplement: Supplementary file 1 [file cells-09-02069-s001.pdf]

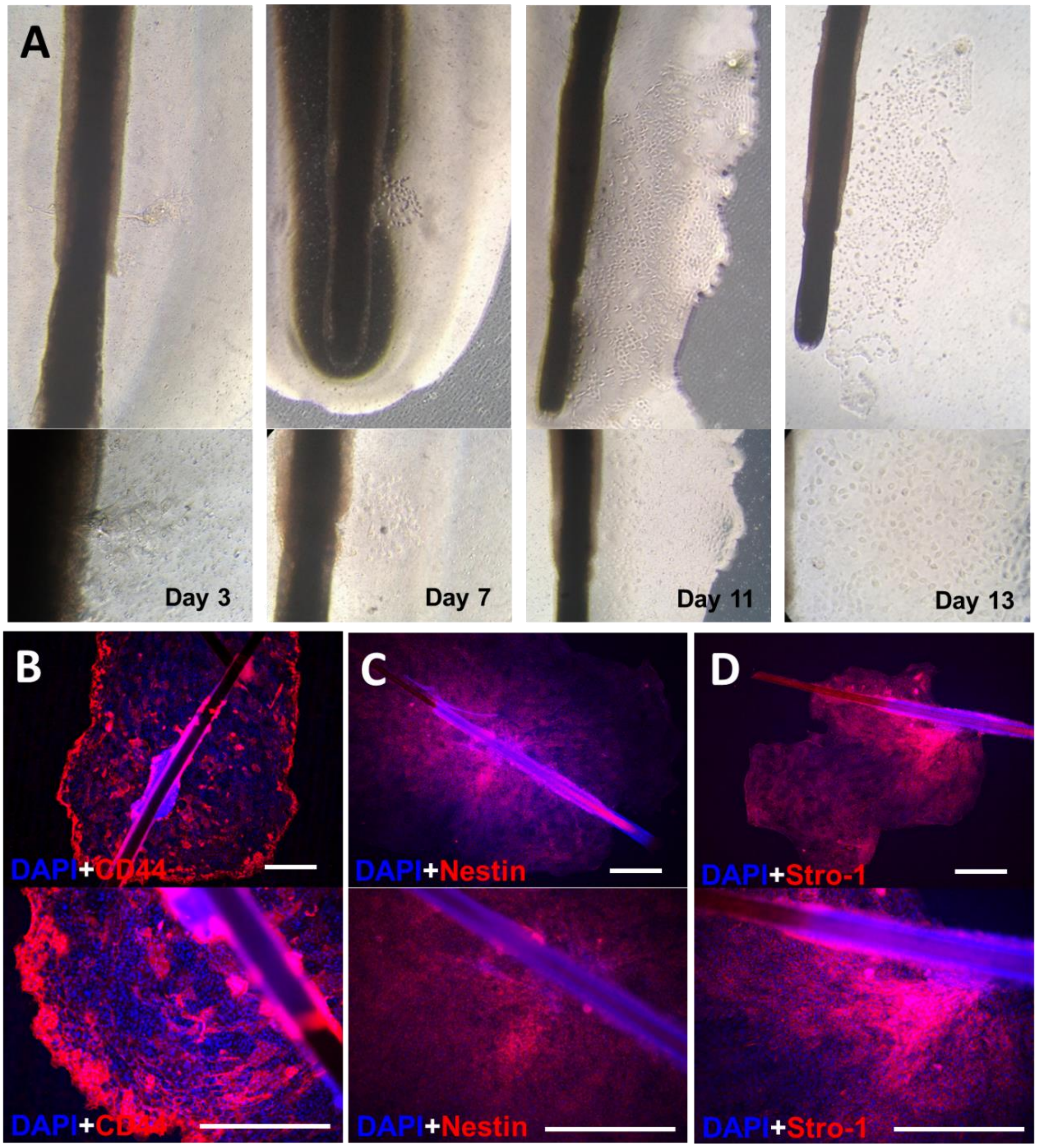

Figure S1. Outgrowth process of MSCORS from the ORS and characterization of ORS cell monolayer. (**A**): Chronologic demonstration of hair follicle ORS cell outgrowth and migration onto the substrate membrane and formation of the cell monolayer. ORS cells outgrew and migrated out of the hair follicle ORS and formed a cell monolayer, which were immunostained using several stem cell biomarkers including CD44 (B), Nestin (C) and Stro-1 (D). Cells at the outer ORS edge intensively expressed CD44. The original sprouting point of cell outgrowth and migration route was visible and highlighted by Nestin and Stro-1 staining. Scale bar: 250 μm.

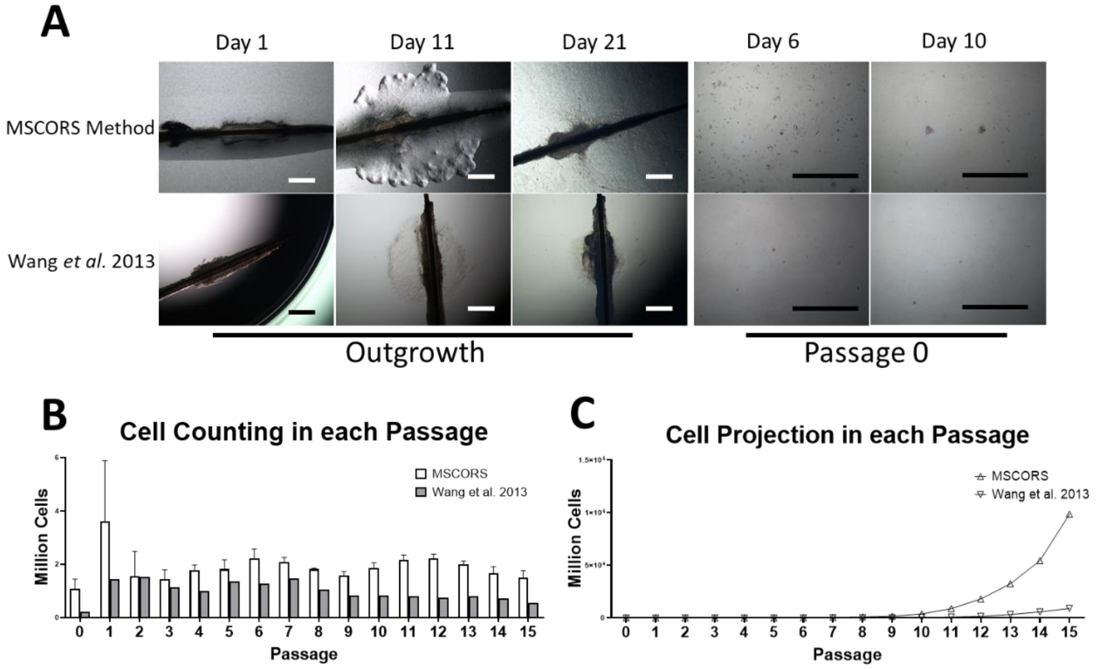

Figure S2. Comparative experiment of MSCORS isolation method vs. outgrowth methods in prior art literature. In these studies of "Li et al. 2015", "Zhang et al. 2013"and "Wang et al. 2013", plucked hair follicles from the intact human hair follicles and adherence-outgrowth method for isolating hair follicle stem cells are shown. All three prior art studies were published by a single group. In the studies described herein, cultivation of the MSCs from plucked hair follicles by an outgrowth onto cell culture plastic in submerged conditions was technically compared to the MSCORS method based on the adherence and outgrowth of the MSCs from the follicle onto the artificial mesh support in liquid-air-interface conditions, including subsequent cultivation and harvesting. On day 21, 32 or 45, respectively, hair follicles produced a confluent cell monolayer on the Transwell mesh as expected in the routinely optimized MSCORS method. The prior art methods brought about outgrowing cell monolayers from 2, 1, and 4 hair follicles upon "Li et al. 2015", "Zhang et al. 2013" and "Wang et al. 2013" method, respectively. (**A**) Representative photos of cell outgrowth on day 1, 11 and 21 in MSCORS and the three prior art methods. MSCORS method yielded a larger area of the cell outgrowing monolayer compared to the prior art method. (**B**) After day 21, cells that migrated from the ORS were subcultured onto the cell culture vessel 2D surface. After subculture from the outgrowth monolayer cultivation, MSCs were expanded. Due to the accumulated number of cells in aforementioned steps, the seeding number of MSCORS was favorable for proliferation whereas the cells in the prior art reached low accumulated and hereby also seeding number and further divided slowly. By day 32, the MSCORS were expanded into 4 × T75 flasks of cumulative surface of 300 cm^2 with 95% confluence as expected, whereas the prior art method of Wang et al. 2013 yielded 1 well 6-well plate with cells reaching 50% confluence. MSCORS method provided higher numbers of the initially seeded cells, which immediately attached onto the cell culture vessels and proliferated rapidly. Cells from prior art methods were low in numbers already in the starting seeding amount. The cells cultivated upon MSCORS and "Wang et al. 2013" methods were expanded in the course of 15-passages. (**B**) Empirical cell count in each passage of the continuous cultivation of MSCORS and prior art methods. (**C**) Projected estimation of cell yield of MCORS and "Wang et al. 2013" upon every passage. After 35 days upon hair plucking, MSCORS method with 45 plucked hairs yielded 4.3171 × 10^6 cells in P0, whereas Wang et al. 2013 yielded 2.108 × 105 cells. The number of cells obtained by MSCORS method was 11-fold higher in P15 than that generated by the prior art method of "Wang et al. 2013".

Table S1. Medium compositions for cell culture.

MSCORS Washing Medium	DMEM (Low Glucose)<br>200 U/mL Penicillin<br>200μg/mL Streptomycin<br>100 μg/mL Gentamycin<br>20 μg/mL Amphotericin B
MSCORS Isolation Medium	DMEM (Low Glucose)<br>10% Fetal Bovine Serum<br>1% ITS Premix<br>10ng/mL bFGF<br>20ng/mL rhEGF<br>2mM L-Glutamine<br>1% Pen/Strep (Penicillin 100U/mL, Streptomycin 100μg/mL)
MSCORS/ADMSC Cultivation Medium	DMEM (Low Glucose)<br>10% Fetal Bovine Serum<br>2mM L-Glutamine<br>1% Pen/Strep (Penicillin 100U/mL, Streptomycin 100μg/mL)
MSCORS Chondrogenic Medium	DMEM (Low Glucose)/F12<br>1% Human Serum<br>1% ITS Premix<br>2mM L-Glutamine<br>10ng/mL TGF-β1<br>10ng/mL BMP-4<br>50ug/mL Ascorbic Acid<br>50ug/mL Na Pyruvate<br>1% Non-essential AA
MSC Osteogenic Medium	DMEM (Low Glucose)<br>10% Fetal Bovine Serum<br>2mM L-Glutamine<br>200nM Dexamethasone<br>50ug/mL Ascorbic Acid<br>10mM β-glycerophosphate
MSCORS Endothelial Medium	DMEM (Low Glucose)<br>5% Fetal Bovine Serum<br>2mM L-Glutamine<br>0.05mM 2-Mercaptoethanol<br>30ng/mL VEGF<br>5ng/mL BMP-4
MSCORS Smooth Muscle Medium	DMEM (Low Glucose)<br>10% Fetal Bovine Serum<br>2mM L-Glutamine<br>10ng/mL TGFβ-1

Table S2. Primer sequences.

Gene	Primer sequence
CD73 for	(CTTTCGCACCCAGTTCACG),
CD73 rev	(TCGTTGGTGTGCAAAATCGT),
CD90	QT00023569, QuantiTect Primer Assays, Qiagen, Hilden, Germany
CD105	QT00013335, QuantiTect Primer Assays, Qiagen, Hilden, Germany
NES for	(CTGCGGGCTACTGAAAAGT),
NES rev	(GTTTGCAGCCGGGAGTTC),
CD45 for	(CTTAGGGACACGGCTGACTT),
CD45 rev	(TGCCCTGTCACAAATACTTCTG),
HTRP for	(ACCACCGTGTGTTAGAAAAGT),
HTRP rev	(CTGCTGACAAAGATTCACTGGT),
